# *Salvia officinalis* flowers extract ameliorates liver and kidney injuries induced by simultaneous intoxication with ethanol/castor oil

**DOI:** 10.14814/phy2.15854

**Published:** 2023-11-13

**Authors:** Saber Jedidi, Kais Rtibi, Houcine Selmi, Foued Aloui, Hichem Sebai

**Affiliations:** ^1^ Laboratory of Functional Physiology and Valorization of Bio‐Resources University of Jendouba, Higher Institute of Biotechnology of Béja Béja Tunisia; ^2^ Laboratory of Sylvo‐Pastoral Resources Institution of Agricultural Research and Higher Education (IRESA), University of Jendouba, Sylvo‐Pastoral Institute of Tabarka Tabarka Tunisia

**Keywords:** ethanol/castor oil, Hepato‐ and nephroprotective, inflammation, oxidative stress, *Salvia officinalis*

## Abstract

The current study investigated the possible mechanisms of aqueous extract *Salvia officinalis* flowers (SF‐AE) and its protective effects against hepatorenal toxicities produced by simultaneous acute administration of ethanol (EtOH)/castor oil (CO). Healthy male rats (*N* = 50) were separated into five equal groups: control, Ethanol (EtOH) + Castor oil (CO), doses of increasing orders of SF‐AE (50, 100, and 200 mg/kg, b.w., p.o.) during 15 days. Liver and kidney injuries were induced by EtOH (4 g/kg, b.w., p.o.) combined with CO (5 mL/kg, b.w., p.o.). Compared to the control group, SF‐AE pretreatment protected against simultaneous administration of EtOH and CO‐caused serious histological alterations in liver and kidney tissues. SF‐AE also reversed liver and kidney biochemical parameters and lipid profile alterations. More importantly, SF‐AE significantly reduced the malondialdehyde (MDA) level and counteracted the depletion of both enzymatic and non‐enzymatic antioxidants. SF‐AE also prevents against inflammation induced by EtOH combined with CO, expressed by the rise of inflammation biomarkers (C‐reactive protein: CRP and alkaline phosphatase: ALP). Additionally, combined EtOH intoxication and CO poisoning exerted an increase in H_2_O_2_, free iron and calcium levels. Impressively, SF‐AE treatment regulated levels of these studied intracellular mediators in a dose‐dependent manner. In conclusion, SF‐AE can potentially improve liver and kidney injuries associated with biochemical parameter deregulations, possibly by controlling oxidative stress and inflammation.

## Graphical abstract

Hepato‐nephroprotective actions of *Salvia officinalis* aqueous extract against simultaneous intoxication with ethanol/castor oil.

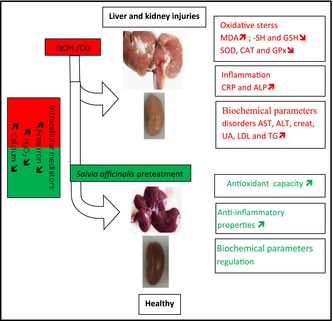

## 1 INTRODUCTION

Liver and kidney are two vital organs in the human system. They can be affected by various factors such as viral infections, high alcohol and drugs utilization (Manzoor et al., 2018). However, the consumption of ethanol is an agent responsible for many pathologies, such as hepatotoxicity, nephrotoxicity, and cardiovascular diseases (Li et al., 2023). In this respect, the World Health Organization (WHO) has counted that about 76 million people are currently alcohol‐dependent and suffer from hepatorenal damage linked to excessive alcohol consumption (WHO, 2019). Furthermore, excessive alcohol consumption has been shown to be considered the number three factor that causes death in modern countries (Weinberger et al., 2019).

The metabolism of alcohol essentially takes place by oxidation to acetaldehyde and then to acetate under the effect of two enzymes, alcohol dehydrogenase (ALD) and aldehyde dehydrogenase (ADH). The microsomal ethanol oxidation system (MEOS/CYP2E1) and catalase are other oxidation pathways. It has been reported that a large proportion of the alcohol consumed (80%) was transformed in the liver tissues; unfolding the intimate relationship between alcoholism and liver disease (Dali‐Youcef & Schlienger, 2012; Liu et al., 2015).

Regarding castor oil, it is generally consumed thanks to the purgative and laxative properties. It is metabolized under the action of pancreatic lipases into two active principles: ricinoleic acid and glycerol. The ricinoleic acid would increase intestinal peristalsis, acceleration of gastrointestinal transit, alteration of the intestinal membrane, and intense purification accompanied by losses of water and electrolytes, and it inhibits the absorption of fat‐soluble vitamins (Rtibi et al., 2017; Tunaru et al., 2012). It may express abdominal pain and cramps, colic, nausea and vomiting. Castor oil is not recommended during pregnancy and breastfeeding; it can induce uterine contractions (Eid & Jaradat, 2020; Tunaru et al., 2012). Furthermore, the toxicity of castor oil is due to the presence of ricin, a highly toxic protein found naturally in castor beans. It was detected in the order of 0.43 mg/10 mL of castor oil (Ratzan & Parker, 2000). It has been determined that a single ricin A chain (RTA) can inactivate 1500 ribosomes/min and that a single ricin molecule is capable of killing a eukaryotic cell (Franz & Jaax, 1997). Ricin is the most commonly used agent for biocrimes (Becher et al., 2007). It is characterized by a high bioavailability, indeed it has been suggested that ricin, a few hours after ingestion, makes its way through the blood to body tissues. Long‐term use of castor oil can lead to fluid and electrolyte loss that can lead to death (Riaz & Farrukh, 2014).

On the contrary, digestive pathologies such as peptic ulcer combined with secretory diarrhea can lead to leakage of fluids and electrolytes, blood, and proteins into the intestinal lumen.

The increase in inflammatory cells leads to the formation of a diffusion barrier and the release of mediators, which alter enzymes in the intestinal brush border and Small bowel villous atrophy. These conditions contributed to the increase of nutrient malabsorption and gastrointestinal transit acceleration (Jedidi et al., 2022a; Zoran, 2008). The malabsorption can induce hepatic and renal pathologies. In this context, clinical examination revealed the increase of transaminase activities and renal function parameters alteration (Drebber et al., 2005; Jedidi et al., 2019).

In addition, alcohol intoxication combined with castor oil poisoning can lead to overproduction of inflammatory cytokines, interleukins, and reactive oxygen species (ROS) within target organs. These factors lead to tissue damage and a redox imbalance state (Jedidi et al., 2022a; Sahin et al., 2018). Overproduction of ROS induced with alcohol/castor oil produces lipid peroxidation and it generates cellular alterations that lead to liver/renal toxicities (Amujiri et al., 2021; Farzaei et al., 2018).

Liver and kidney transplants and the use of marketed drugs, such as naltrexone, nalmefene, baclofen, or acamprosate were considered emerging surgical procedures and/or treatment agents (O'Leary et al., 2016; Palpacuer et al., 2018). However, the toxic effect of drugs and the lack of sufficient donors play limiting factors against these therapeutic and surgical interventions. Consequently, alternative solutions are needed to treat hepatorenal lesions (Cook et al., 2016; O'Leary et al., 2016).

In this respect, several research studies have examined the therapeutic potential to prevent/treat these pathologies and in particular the consumption of natural plant‐based phytonutrients. Recently, increasing attention has been drawn to researching the hepato‐ and nephroprotective properties of herbal nutraceuticals due to their minimal toxicity and numerous pharmacological properties (Kang et al., 2021; Xiong & Guan, 2017).

*Salvia officinalis* L. (Lamiaceae family), is widely used in ethno‐medicine. However, the ethnopharmacological survey carried out in Northwestern Tunisia revealed that sage is indeed traditionally used to treat/prevent several pathologies, in particular those of the digestive tract (Jedidi et al., 2018).

Thanks to its richness in bioactive molecules, sage is endowed with several biological properties such as antidiabetic and antibacterial (Al‐Mijalli et al., 2022), antiviral (Abou Baker et al., 2021), and antiulcer (Jalalipour et al., 2022) activities. In addition, we recently demonstrated that the flowers decoction extract exerted hepato‐ and nephroprotective activities against alcohol intoxication alone (Jedidi et al., 2022b).

Hence, the current study explored the hepatonephroprotective effects of SF‐AE against acute intoxication of ethanol combined with castor oil.

## 2 MATERIALS AND METHODS

### 2.1 Plant collection, identification, and preparation of flowers aqueous extract

The fresh plant material (flowers of *Salvia officinalis* L.) was collected in Tabarka (Tunisia) in April 2020. The specimens (No. S321) have been deposited with the herbarium of the Higher Institute of Biotechnology of Béja (University of Jendouba‐Tunisia). The washed plant material was dried and then ground into a fine powder.

For the preparation of plant extracts, many test portions of 1 g of powder were freshly macerated in 20 mL of distilled water (1/20; w/v) for 24 h. The products thus obtained were filtered and the filtrates were lyophilized. Finally, the obtained dry residues were weighed and subsequently used for phytochemical and biological analysis.

#### 2.1.1 SF‐AE extract polyphenolic content

The Folin–Ciocalteu method was used to assess the phenolic compound content in the aqueous extract and using gallic acid (GA) as the standard solution. In this method, 0.5 mL of Folin–Ciocalteu solution (1/10), 0.5 mL of sage aqueous extract, and 1 mL of carbonate sodium (7.5%) were mixed and incubated for 1 h at room temperature. Finally, the resulting absorbance was read at a wavelength of 765 nm (Dewanto et al., 2002).

#### 2.1.2 Determination of flavonoids content

The aluminum trichloride (AlCl_3_) method was used to quantify the total flavonoids content in SF‐AE (Yi et al., 2007). Briefly, 1 mL of SF‐AE was added to 1 mL of the AlCl_3_ solution (2%). Fifteen minutes later, absorbance was determined at 510 nm using a UV/visible spectrophotometer.

#### 2.1.3 SF‐AE extract condensed tannin content

The evaluation of condensed tannins content in the plant extract was calculated according to the vanillin method in an acid medium. The result was determined using colorimetric procedure and catechin (C) as the standard solution (Price et al., 1978).

#### 2.1.4 In vitro antioxidant activity

In this method, 1000 μL of serial dilution of aqueous extract of SF‐AE (10, 50, 100, 150, 200, 300, 350 and 400 μg/mL) and 1 mL of radical ethanol solution of 2, 2‐diphenyl‐1‐picrylhydrazyl (DPPH^●^) (2.4 mg /100 mL) were mixed. After incubation at room temperature for 30 min, the absorbance of the resulting mixture was read at a wavelength of 517 nm by colorimetric procedure (Ben Ammar et al., 2009). In addition, BHT (butylated hydroxytoluene) was used as a reference molecule in the same concentration as the test SF‐AE.

### 2.2 Animals and experimental design

Fifty male *Wistar* rats, weighing 210 ± 20 g, were randomly divided into 5 groups (10 animals each). The rats were kept for 5 days to adapt to the temperature, food, and water used throughout the experiment. All rats were housed in propylene cages at a temperature of 22 ± 2°C with a 12/12 dark/light cycle. Standard rat pellets and potable tap water were used for all groups. All maintenance and sacrifice procedures were used following the local ethics committee of Tunis University of the use and care of animals and in accordance with the NIH recommendation. The protocol was approved by the “Comité d'Ethique Bio‐medicale (CEBM)” (JORT472001) of the “Institut Pasteur de Tunis.”

#### 2.2.1 Hepatonephrotoxicity induced by simultaneous intoxication with EtOH/CO

The rats were divided into five groups of ten animals each and were pretreated daily at 8 am for 15 days, as follows:

- Group 1 served as control untreated group, received distilled water (5 mL/kg, b.w., p.o.)
- Group 2 served as positive control and received distilled water (5 mL/kg, b.w., p.o.)
- The other three groups were pretreated daily with increasing doses of freshly prepared SF‐AE (50, 100, and 200 mg/kg, b.w., p.o.). Preliminary experiment indicated that 50, 100, and 200 mg/kg SF‐AE were the lowest doses that give a significant protective effect and did not cause any sign of toxicity during the 15 days of treatment.

The rats were fasted for 18 h before the last administration of SF‐AE. After 60 min, each animal except the rats of the first group received EtOH (4 g/kg^−1^, b.w., p.o.) orally and after 30 min, they were intoxicated with castor oil (5 mL/kg, b.w., p.o.). Two hours later, animals were anesthetized by intraperitoneal administration of sodium pentobarbital (40 mg/kg, b.w.) and sacrificed by decapitation.

Blood was collected in heparinized tubes and the plasma thus obtained was stored at −20°C. The liver and kidney tissues were removed, weighed and dissected into small pieces.

The samples were ground in phosphate buffer (19.5 mM KH_2_PO_4_/30.5 mM K_2_HPO_4_, pH 7.4) on ice. The products thus obtained were centrifuged at 10,000 ***g*** during 15 min. Finally, the homogenate was stored at −80°C for biochemical analysis.

Concerning the microscopic preparation, tissue fragments were fixed in a buffered neutral formalin solution (10%). Histology was determined according to the method of Behmer & Tolosa (2003).

#### 2.2.2 Determination of hepatorenal functions and metabolic parameters

Serum alanine aminotransferase (ALT, Ref 11046), aspartate aminotransferase (AST, Ref 10049), lactate dehydrogenase (LDH, Ref 13033), urea (Ref 30016), creatinine (Ref 25036), uric acid (Ref 15013), blood urea nitrogen, total cholesterol (Ref 21021), HDL (Ref 22011) and LDL‐cholesterol (Ref 24015) and triglycerides (Ref 29058) were measured by colorimetric method using commercially available diagnostic kits (Biomaghreb).

#### 2.2.3 Determination of liver toxicity biomarkers

Commercial reagent kits purchased from Biomaghreb were used to determine the levels of albumin (Ref 16010) and direct bilirubin (Ref 17017) in plasma.

#### 2.2.4 Assessment of anti‐inflammatory activity of SF‐AE

Commercial reagent kits purchased from Biomaghreb were used for the inflammation biomarkers assay such as C‐reactive protein (Ref 45027) level and alkaline phosphatase (Ref 13033) activity.

### 2.3 Effects of SF‐AE on oxidative stress induced by EtOH/CO

#### 2.3.1 Liver and kidney tissues malondialdehyde (MDA) levels

The lipid peroxidation in hepatic and renal tissues was determined by measuring the level of MDA, using the double heating method (Draper & Hadley, 1990). Briefly, aliquots of homogenates were mixed with a butylated hydroxytoluene (BHT)‐trichloroacetic acid (TCA) solution containing BHT (1%) dissolved in 20% (w/v) TCA. The product was centrifuged at 1000 ***g*** for 5 min at 4°C. The supernatant was mixed with a solution containing (0.5 N HCl, 120 mM TBA buffer buffered in 26 mM Tris), then heated at 80°C for 10 min. MDA levels were calculated using a molar extinction coefficient of MDA‐TBA complex which is equal to 1.56 × 105 M/cm.

#### 2.3.2 Determination of hydrogen peroxide level

The H_2_O_2_ concentrations in liver and kidney tissues were determined according to the method previously described by Dingeon et al. (1975). In fact, in the presence of phenol, hydrogen peroxide, and peroxidase, 4‐aminoantipyrine available in the reaction medium, lead to the formation of quinoneimine with pink color detected at 505 nm. The standard curve is made with hydrogen peroxide at different concentrations.

#### 2.3.3 Assessment of superoxide dismutase (SOD) activities

The spectrophotometric method was used to evaluate the SOD activity in hepatic and renal tissues using the epinephrine/adenochrome system (Misra & Fridovich, 1972). At basic pH, the superoxide anion (O_2_^•−^) induces the autoxidation of epinephrine to adenochrome. In this reaction, SOD competes by inducing the formation of hydrogen peroxide (H_2_O_2_) molecules from O_2_^•−^, catalase catalyzes the formation of water from H_2_O_2_ to prevent the reformation of O_2_^•−^. The enzyme extract was added to 2 mL of reaction mixture containing 10 μL of bovine catalase (CAT, 0.4 U/mL), 20 μL of epinephrine (5 mg/mL), and 62.5 mM of buffer sodium carbonate/bicarbonate (pH 10.2). Changes in absorbance were evaluated at 480 nm.

#### 2.3.4 Liver and renal tissues catalase (CAT) activities

The activity of CAT was assessed by measuring the rate of hydrogen peroxide disappearance by spectrophotometry at 240 nm, which will be degraded into H_2_O and O_2_ (Aebi, 1984). The reaction mixture contained 33 mM H_2_O_2_ in 50 mM phosphate buffer (pH 7). Thus, the gradual decrease in optical density corresponds to the decomposition of H_2_O_2_ by CAT. The activity of catalase is expressed in μmoles H_2_O_2_/mg protein.

#### 2.3.5 Determination of glutathione peroxidase (GPx) activity

The activity of glutathione peroxidase was quantified according to the method of Flohé and Gunzler (1984). Briefly, 1 mL of reaction mixture containing 0.2 mL of liver and kidney tissue supernatant, 0.2 mL of phosphate buffer (0.1 M pH 7.4), 0.2 mL of GSH (4 mM), and 0.4 mL of H_2_O_2_ (5 mM) was incubated at 37°C during 1 min. The reaction was stopped by the addition of 0.5 mL of TCA (5% w/v). After centrifugation at 1500 ***g*** for 5 min, a 0.2 mL of aliquot from the supernatant was mixed with 0.5 mL of phosphate buffer solution 0.1 M pH 7.4 and 0.5 mL of DTNB (10 mM). The absorbance was read at 412 nm. The GPx activity was expressed in mmol of GSH consumed/min/mg protein.

#### 2.3.6 Liver and kidney tissues groupements sulfhydryls and reduced glutathione levels

The thiol groups (‐SH) content in liver and kidney tissues were determined according to the method of Ellman (1959). Samples were mixed with 800 μL of phosphate buffer (pH 8.2; 0.25 M) and 100 μL of EDTA (20 mM). The mixture is vortexed and the absorbances were measured at 412 nm (A_1_). Then we added 100 μL of DTNB (10 mM) and the reaction mixture was incubated at 37°C for 15 min and a new value (A_2_) was determined. The concentration of thiol groups was calculated by subtraction operation between two absorbances (A_2_ and A_1_) using a molar extinction coefficient of 13.6 × 103 M^−1^ cm^−1^. The results were expressed in nmol of thiol groups/mg proteins.

Liver and kidney tissues reduced glutathione (GSH) levels were carried consistently with method previously described by Sedlak and Lindsay (1968) and using DTNB (0.01 M) as reactive. In fact, 5 mL of supernatant was mixed with 4 mL of distilled water and 1 mL of TCA 50%. The tubes were centrifuged at 1200 ***g*** for 15 min. 2 mL of the product thus obtained are mixed with 4 mL of Tris buffer (0.4 M; pH 8.9). Absorbance was recorded at 412 nm against a blank containing only the buffer.

### 2.4 Determination of free iron and calcium concentrations

Free iron (Fe, Ref 03027) and calcium (Ca, Ref 01023) contents in plasma, liver, and kidney tissues were performed using commercially available diagnostic kits (Biomaghreb). Briefly, the iron dissociated from transferrin‐iron complex by a solution of guanidine acetate and reduced by ascorbic acid reacts with ferrozine to give a pink complex measured at 562 nm. Regarding the calcium level, the Ca^2+^, which precipitates as Ca^2+^ oxalate, forms a complex with the o‐cresolphthalein. Finally, the absorbance was determined at 570 nm.

### 2.5 Determination of total protein content

Total protein (Ref 27016) levels were determined using commercially available diagnostic kits (Biomaghreb).

### 2.6 Statistical analysis

The experimental results are presented as the mean ± SE. Data were analyzed by one‐way analysis of variance (anova) using statistical software SAS (Statistics Analysis System, Version 8.2). The difference is considered significant when the *p* < 0.05.

## 3 RESULTS

### 3.1 Phytochemical compounds and antioxidant capacity of SF‐AE

The results of phenolic compounds and antioxidant capacity were presented in Table 1. We have shown that SF‐AE is characterized by its richness in total polyphenols, flavonoids, and condensed tannins.

**TABLE 1:** Phenolic compounds composition and antioxidant capacity of *Salvia officinalis* flowers aqueous extract (SF‐AE) and Butylated hydroxytoluene (BHT).

Parameters	Contents/IC_50_
Total polyphenols (mg GAE/g DM)	101.37 ± 3.56
Flavonoids (mg QE/g DM)	52.69 ± 2.62
Condensed tannins (mg CE/g DM)	8.76 ± 0.23
DPPH^•^ (IC_50_, μg/mL)	50.18 ± 2.09
BHT (IC_50_, μg/mL)	41.04 ± 1.26

*Note*: Data were presented as mean ± SD (*n* = 3 of independent SF‐AE preparations).

Abbreviations: CE, catechin equivalent; DM, dry matter; GAE, gallic acid equivalent; IC_50,_ Inhibitory concentration 50; QE, Quercetin equivalent; SD, standard deviation.

The DPPH^•^ radical scavenging activity of SF‐AE was evaluated by determining the IC_50_ value (Table 1). The antioxidant activity of SF‐AE extract and butylated hydroxytoluene (BHT) against DPPH assay was tested with concentrations ranging from 10 to 400 μg/mL. The SF‐AE was effective in inhibiting the free radical DPPH^•^ in a dose‐dependent manner. The IC_50_ value of SF‐AE is low (50.18 ± 2.09 μg/mL), but still slightly high when compared to that of BHT (41.04 ± 1.26 μg/mL), used as a reference antioxidant molecule.

### 3.2 Histological changes in liver and kidney

**FIGURE 1:**
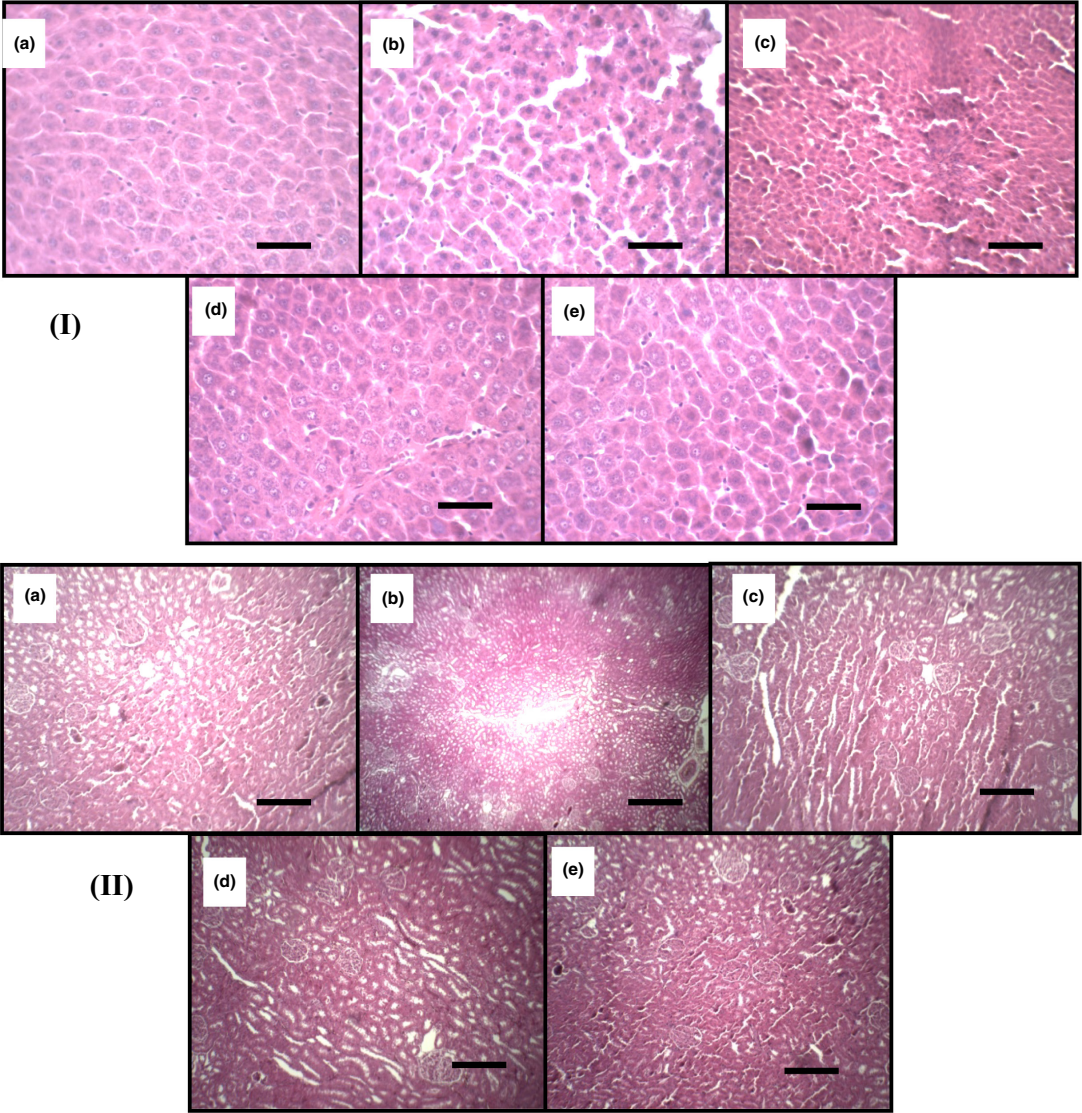
Liver (I) and kidney (II) histological observation showing the protective action of *Salvia officinalis* flowers aqueous extract (SF‐AE) against simultaneous intoxication with ethanol and castor oil (EtOH + CO)‐induced injures in liver and kidney. Animals were treated with various doses of SF‐AE (50, 100, and 200 mg/kg, b.w., p.o.) or vehicle (NaCl 0.9%). (a) H_2_O + NaCl; (b) H_2_O + EtOH+ CO; (c–e) SF‐AE (50, 100, and 200 mg/kg, b.w., p.o., respectively) + EtOH+ CO. H&E, ×200, scale bar = 20 μm.

After treatment, the liver and kidneys were examined histologically to determine if EtOH combined with CO could affect tissue structures. Sections stained with H‐E showed regular normal morphology in the kidney and liver (Figure 1a). In contrast, treatment with EtOH combined with CO negatively affected liver and kidney histological aspect. This intoxication induced focus of necrosis and blood congestion with dilation of the centrilobular veins and edemas are observed in the livers. Distensions of the renal tubules blood congestion and renal parenchyma are also showed in the kidneys (Figure 1b). The SF‐AE treatment protected against alterations in tissue structures. However, SF‐AE reduced liver and kidney edema and inflammation (Figure 1c–e).

### 3.3 SF‐AE regulated liver and kidney weights

**FIGURE 2:**
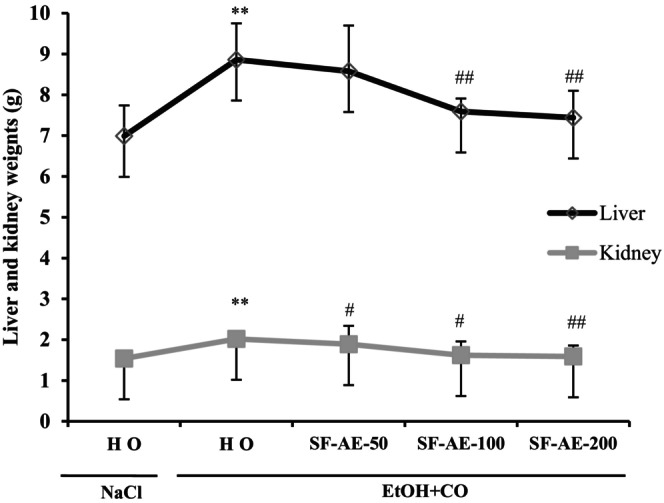
Effect of *Salvia officinalis* flowers aqueous extract (SF‐AE) against simultaneous intoxication with ethanol and castor oil (EtOH + CO)‐induced changes in liver and kidney weights. Animals were pretreated with various doses of SF‐AE (50, 100, and 200 mg/kg, b.w., p.o.) or vehicle (NaCl 0.9%) during 15 days. One hour after, each animal, except group 1 was received EtOH (5 mL/kg, b.w.) and after 30 min castor oil (5 mL/kg, bw., p.o.) by gavage for 2 h. The data are expressed as mean ± SD (*n* = 10). **p < 0.01 compared to control group; ^#^ *p* < 0.05 and ^##^ *p* < 0.01 compared to EtOH + CO group.

Liver and kidney weights were determined after 15 days of treatment. Rats intoxicated with EtOH + CO showed a significant increase in liver and kidney weights (Figure 2). This increase signaled the induction of hepatorenal injuries in rats. However, rats pretreated with SF‐AE showed weights close to those of the control.

### 3.4 SF‐AE restored hepatic function deregulations and non‐enzymatic biomarkers toxicity

Enzyme activities (AST, ALT, and LDH) were determined to assess liver function. In this respect, simultaneous intoxication with EtOH/CO in healthy rats altered enzyme activities, compared to the untreated group, thus confirming acute liver toxicity. Pretreatment of SF‐AE partially regulated these biomarkers activities relative to the EtOH + CO group (Table 2).

**TABLE 2:** Effect of *Salvia officinalis* flowers aqueous extract (SF‐AE) against simultaneous intoxication with ethanol and castor oil (EtOH + CO)‐induced hepatic function deregulations (AST, ALT, and LDH) and non‐enzymatic biomarkers toxicity (plasmatic albumin and direct bilirubin levels).

Groups and parameters	Control	EtOH + CO	EtOH + CO + SF‐AE‐50	EtOH + CO + SF‐AE‐100	EtOH + CO + SF‐AE‐200
AST (UI/L)	99.93 ± 4.78	168.24 ± 15.67*	154.38 ± 13.75#	143.52 ± 11.39#	125.76 ± 8.81#
ALT (UI/L)	58.41 ± 4.52	89.83 ± 7.98*	80.34 ± 5.54#	76.66 ± 6.55#	64.32 ± 4.43#
LDH (U/L)	55.12 ± 3.26	123.72 ± 12.33*	117.66 ± 10.44#	98.18 ± 8.54#	61.98 ± 6.88#
Albumin (mg/L)	26.23 ± 4.51	11.65 ± 3.37*	15.87 ± 3.98#	16.07 ± 4.12#	20.43 ± 5.55#
Direct bilirubin (mg/g)	0.14 ± 0.08	0.06 ± 0.01*	0.07 ± 0.03	0.09 ± 0.03#	0.11 ± 0.09#

*Note*: Animals were pretreated with various doses of SF‐AE (50, 100, and 200 mg/kg, b.w., p.o.) or vehicle (NaCl 0.9%) during 15 days. One hour after, each animal, except group 1 was received EtOH (5 mL/kg, b.w.) and after 30 min castor oil (5 mL/kg, bw., p.o.) by gavage for 2 h. The data are expressed as mean ± SD (*n* = 10).

* *p* < 0.05 compared to control group.

^#^ *p* < 0.05 compared to EtOH + CO group.

We also tested other liver biological markers (albumin and direct bilirubin). As shown in Table 2, we demonstrated a decrease of albumin and direct bilirubin content in the EtOH + CO group as opposed to the control group. However, *Salvia officinalis* flowers aqueous extract preserved these biomarkers to near normal values.

### 3.5 Assessment of renal function

On the contrary, we evaluated indicators of renal toxicity, such as creatinine, uric acid and urea. The results showed the rise of urea and creatinine contents, and on the contrary, a decrease uric acid in the EtOH/CO group (Table 3). In addition, the obtained results revealed the decrease of blood urea nitrogen in the EtOH + CO group compared to the untreated group. However, SF‐AE pretreatment, during 15 days, exerted the preservation of these indicators when compared to EtOH + CO group.

**TABLE 3:** Effect of *Salvia officinalis* flowers aqueous extract (SF‐AE) against simultaneous intoxication with ethanol and castor oil (EtOH + CO)‐induced changes renal function.

Groups and parameters	Control	EtOH + CO	EtOH + CO + SF‐AE‐50	EtOH + CO + SF‐AE‐100	EtOH + CO + SF‐AE‐200
Creatinine (μmol/L)	30.19 ± 1.43	67.47 ± 3.87*	53.62 ± 1.89#	47.23 ± 2.63#	43.53 ± 4.12#
Urea (mmol/L)	7.92 ± 1.09	10.34 ± 2.16*	10.21 ± 1.23	8.64 ± 1.02#	8.23 ± 1.27#
Uric acid (mmol/L)	0.24 ± 0.05	0.14 ± 0.03*	0.16 ± 0.08#	0.18 ± 0.02#	0.21 ± 0.05#
Blood urea nitrogen (mmol/L)	3.7 ± 0.51	5.01 ± 1.01*	4.77 ± 0.57	4.03 ± 0.47#	3.84 ± 0.59#

*Note*: Animals were pretreated with various doses of SF‐AE (50, 100, and 200 mg/kg, b.w., p.o.) or vehicle (NaCl 0.9%) during 15 days. One hour after, each animal, except group 1 was received EtOH (5 mL/kg, b.w.) and after 30 min castor oil (5 mL/kg, bw., p.o.) by gavage for 2 h. The data are expressed as mean ± SD (*n* = 10).

* *p* < 0.05 compared to control group.

^#^ *p* < 0.05 compared to EtOH + CO group.

### 3.6 SF‐AE restored plasmatic lipid profile

In this investigation, the lipid profile study showed that the administration of EtOH combined with CO caused an increase in the levels of total cholesterol (TC), triglycerides (TG), and LDL‐cholesterol, and on the contrary, a decrease in HDL cholesterol. However, pretreatment with SF‐AE dose‐dependently corrected all these deregulations (Table 4).

**TABLE 4:** Effect of *Salvia officinalis* flowers aqueous extract (SF‐AE) against simultaneous intoxication with ethanol and castor oil (EtOH + CO)‐induced alterations of total cholesterol, HDL and LDL‐cholesterol and triglycerides concentrations in rats.

Groups and parameters	Control	EtOH + CO	EtOH + CO + SF‐AE‐50	EtOH + CO + SF‐AE‐100	EtOH + CO + SF‐AE‐200
TC (mmol/L)	1.15 ± 0.12	2.12 ± 0.18*	2.02 ± 0.15	1.87 ± 0.09	1.23 ± 0.04
HDL‐C (mmol/L)	0.39 ± 0.05	0.76 ± 0.1*	0.68 ± 0.08#	0.48 ± 0.04#	0.42 ± 0.04#
LDL‐C (mmol/L)	0.28 ± 0.02	0.97 ± 0.4*	0.80 ± 0.17#	0.55 ± 0.12#	0.47 ± 0.03#
Triglycerides (mmol/L)	0.63 ± 0.18	1.27 ± 0.51	0.97 ± 0.14	0.84 ± 0.13	0.74 ± 0.07

*Note*: Animals were pretreated with various doses of SF‐AE (50, 100, and 200 mg/kg, b.w., p.o.) or vehicle (NaCl 0.9%) during 15 days. One hour after, each animal, except group 1 was received EtOH (5 mL/kg, b.w.) and after 30 min castor oil (5 mL/kg, bw., p.o.) by gavage for 2 h. The data are expressed as mean ± SD (*n* = 10).

* *p* < 0.05 compared to control group.

^#^ *p* < 0.05 compared to EtOH + CO group.

### 3.7 Anti‐inflammatory capacity of SF‐AE

Ethyl intoxication combined with castor oil induced a rise of CRP level and ALP activity. *Salvia officinalis* flowers aqueous extract subacute pretreatment significantly and in a dose‐related manner excreted protection against the alteration in these studied inflammatory biomarkers (Table 5).

**TABLE 5:** Subacute effect of *Salvia officinalis* flowers aqueous extract (SF‐AE) on plasmatic CRP level and ALP activity induced by simultaneous intoxication with ethanol and castor oil (EtOH + CO) in rats.

Groups and parameters	Control	EtOH + CO	EtOH + CO + SF‐AE‐50	EtOH + CO + SF‐AE‐100	EtOH + CO + SF‐AE‐200
CRP (μg/mL)	32.45 ± 4.12	468.14 ± 21.45*	402.75 ± 32.07#	222.09 ± 14.83#	111.52 ± 8.45#
ALP (U/L)	38.54 ± 4.23	170.45 ± 17.23*	167.28 ± 9.18	150.68 ± 10.17#	108.54 ± 6.19#

*Note*: Animals were pretreated with various doses of SF‐AE (50, 100, and 200 mg/kg, b.w., p.o.) or vehicle (NaCl 0.9%) during 15 days. One hour after, each animal, except group 1 was received EtOH (5 mL/kg, b.w.) and after 30 min castor oil (5 mL/kg, bw., p.o.) by gavage for 2 h. The data are expressed as mean ± SD (*n* = 10).

Abbreviations: ALP, alkaline phosphatase; CRP, C‐reactive protein.

* *p* < 0.05 compared to control group.

^#^ *p* < 0.05 compared to EtOH + CO group.

### 3.8 SF‐AE restored redox status parameters

#### 3.8.1 SF‐AE protects liver and kidney tissues against EtOH + CO lipoperoxydation

**FIGURE 3:**
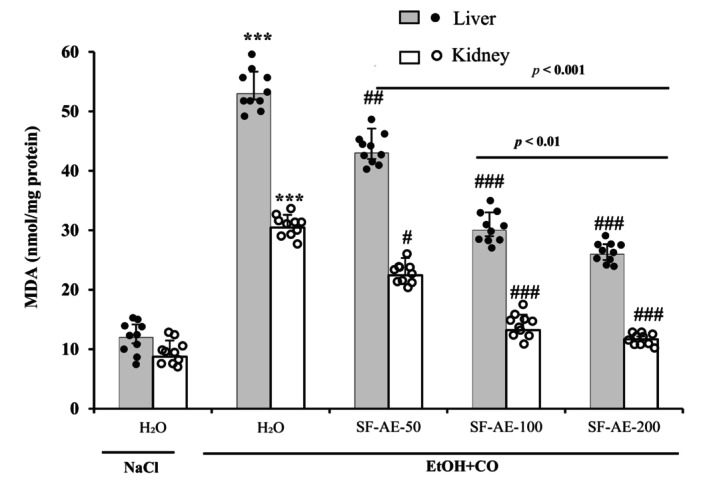
Effect of *Salvia officinalis* flowers aqueous extract (SF‐AE) against simultaneous intoxication with ethanol and castor oil (EtOH + CO)‐induced changes in liver and kidney malondialdehyde (MDA) levels in rats. Animals were pretreated with various doses of SF‐AE (50, 100, and 200 mg/kg, b.w., p.o.) or vehicle (NaCl 0.9%) during 15 days. One hour after, each animal, except group 1 was received EtOH (5 mL/kg, b.w.) and after 30 min castor oil (5 mL/kg, bw., p.o.) by gavage for 2 h. The data are expressed as mean ± SD (*n* = 10). ***p < 0.001 compared to control group; ^#^ *p* < 0.05, ^##^ *p* < 0.01 and ^###^ *p* < 0.001 compared to EtOH + CO group. The lines presented a significant difference between the two groups shown.

Lipo‐peroxidation was assessed by measuring the concentration of malondialdehyde (MDA) in hepatic and renal tissues. Figure 3 illustrates the variation of MDA levels of all treatment groups. Livers and kidneys treated with EtOH + CO showed an increase of MDA levels compared to untreated groups. This increase reflects the damage in the cell membrane exerted by the co‐administration of EtOH + CO. Pretreatment with sage aqueous extract regulated the lipid peroxidation.

#### 3.8.2 SF‐AE regenerates antioxidant enzymes

**FIGURE 4:**
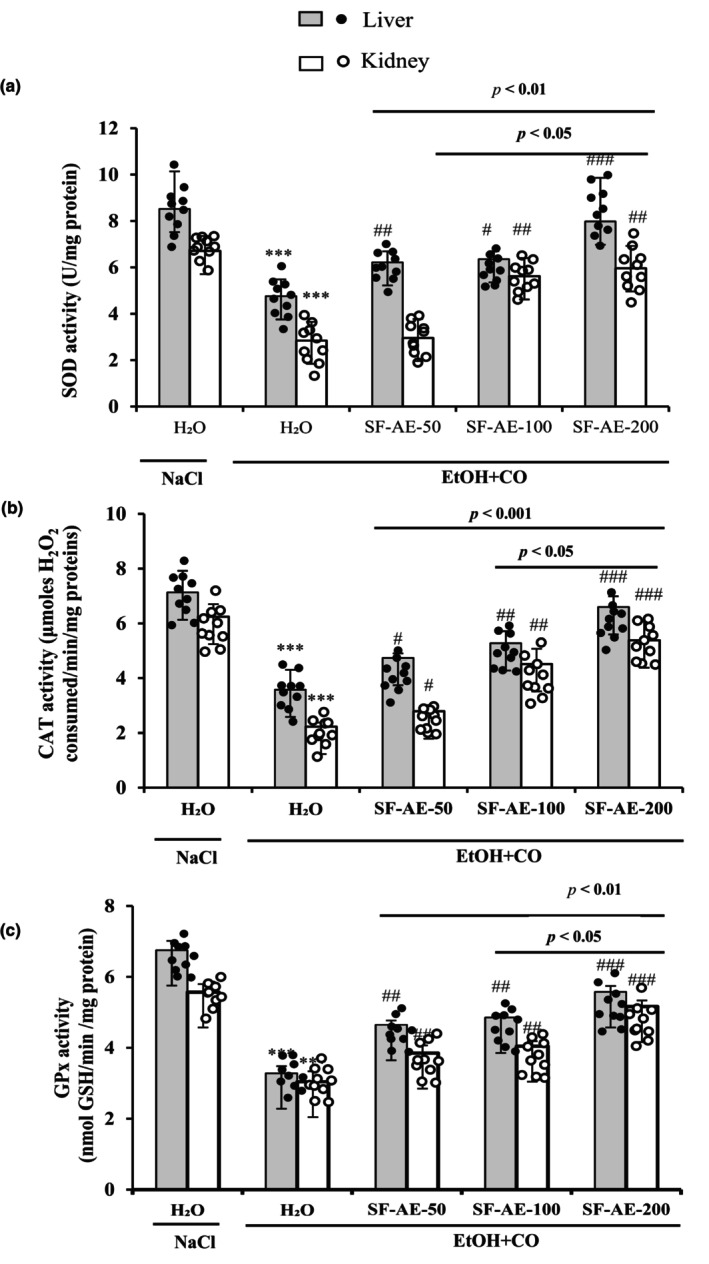
Effect of *Salvia officinalis* flowers aqueous extract (SF‐AE) against simultaneous intoxication with ethanol and castor oil (EtOH + CO)‐induced deregulations in liver and kidney antioxidant enzymes activities: SOD (a) CAT (b) and GPx (c). Animals were pretreated with various doses of SF‐AE (50, 100, and 200 mg/kg, b.w., p.o.) or vehicle (NaCl 0.9%) during 15 days. One hour after, each animal, except group 1 was received EtOH (5 mL/kg, b.w.) and after 30 min castor oil (5 mL/kg, bw., p.o.) by gavage for 2 h. The data are expressed as mean ± SD (*n* = 10). ***p < 0.001 compared to control group; ^#^ *p* < 0.05, ^##^ *p* < 0.01 and ^###^ *p* < 0.001 Compared to EtOH + CO group. The lines presented a significant difference between the two groups shown.

On the contrary, we evaluated the enzymatic antioxidant activities. EtOH combined with CO intoxication induce a significant depletion (*p* < 0.05) of SOD (Figure 4a), CAT (Figure 4b), and GPx (Figure 4c) activities in liver and kidney tissues. Subacute SF‐AE pretreatment (50, 100, and 200 mg/kg, b.w., p.o.) protected the alterations of those enzymatic antioxidant activities (Figure 4).

#### 3.8.3 Regeneration of thiol groups and reduced glutathione levels

**FIGURE 5:**
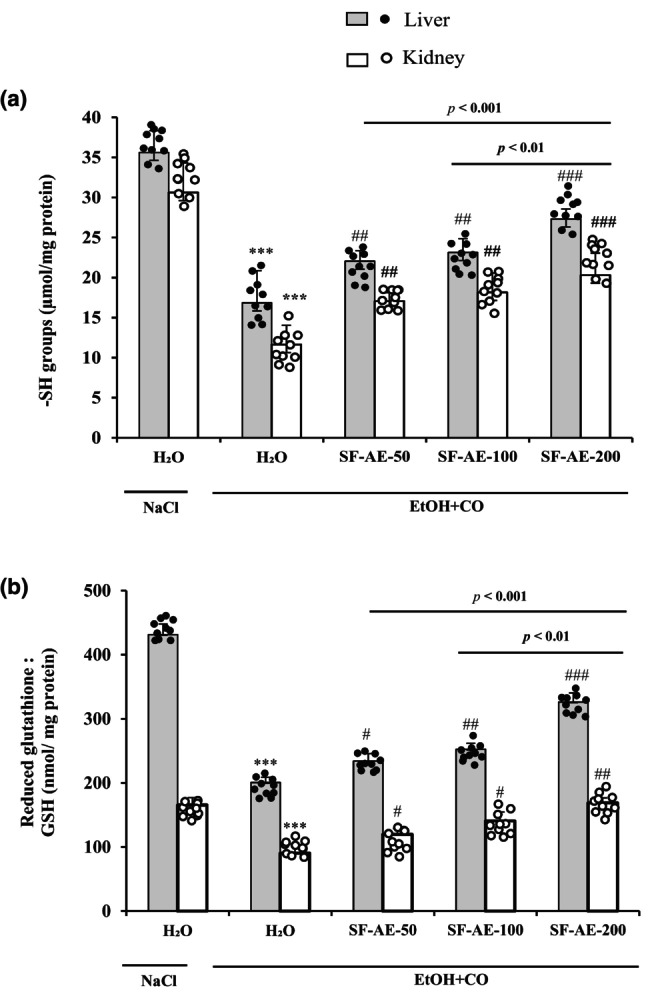
Action of *Salvia officinalis* flowers aqueous extract (SF‐AE) on liver and kidney non‐enzymatic antioxidants levels: sulfhydryl groups (a) and reduced glutathione (b) during simultaneous intoxication with ethanol and castor oil (EtOH + CO)‐induced deregulations. Animals were pretreated with various doses of SF‐AE (50, 100, and 200 mg/kg, b.w., p.o.) or vehicle (NaCl 0.9%) during 15 days. One hour after, each animal, except group 1 was received EtOH (5 mL/kg, b.w.) and after 30 min castor oil (5 mL/kg, bw., p.o.) by gavage for 2 h. The data are expressed as mean ± SD (*n* = 10). ***p < 0.001 compared to control group; ^#^ *p* < 0.05, ^##^ *p* < 0.01 and ^###^ *p* < 0.001 compared to EtOH + CO group. The lines presented a significant difference between the two groups shown.

The changes in the levels of non‐enzymatic antioxidants in liver and kidney tissues were observed in Figure 5. As expected, the oral administration of EtOH combined with CO induces a significant decrease in the level of thiol groups (Figure 5a) and GSH (Figure 5b) in the liver and kidneys. SF‐AE pretreatment significantly and dose‐dependently regenerated the levels of non‐enzymatic antioxidants.

### 3.9 Effect of SF‐AE administration in intracellular mediator's level

The effects of EtOH combined with CO and SF‐AE on hydrogen peroxide, calcium, and free iron in liver and kidney tissues and plasma levels, were performed (Table 6). In the groups that received the alcohol combined with the CO, the levels of these parameters have been increased when compared to the untreated group. More importantly, SF‐AE pretreatment reduced all EtOH/CO‐induced deregulation of intracellular mediators in a dose‐dependent manner.

**TABLE 6:** Effect of *Salvia officinalis* flowers aqueous extract (SF‐AE) against simultaneous intoxication with ethanol and castor oil (EtOH + CO)‐induced alterations in liver, kidney and plasma hydrogen peroxide (H_2_O_2_), free iron and calcium concentrations in rats.

Groups and parameters	Control	EtOH + CO	EtOH + CO + SF‐AE‐50	EtOH + CO + SF‐AE‐100	EtOH + CO + SF‐AE‐200
Hepatic H_2_O_2_ (μmol/mg protein)	2.37 ± 2.54	5.81 ± 4.54*	2.76 ± 3.87#	2.16 ± 3.47#	1.35 ± 2.54#
Renal H_2_O_2_ (μmol/mg protein)	1.08 ± 0.12	3.94 ± 0.65*	2.79 ± 0.47#	1.74 ± 0.08#	1.50 ± 0.07#
Plasma H_2_O_2_ (nmol/L)	1.11 ± 0.08	2.42 ± 0.17*	1.82 ± 0.45#	1.68 ± 0.06#	1.45 ± 0.08#
Hepatic free iron (μmol/mg protein)	69.37 ± 5.87	201.21 ± 12.26*	180.56 ± 9.71#	120.14 ± 10.08#	101.23 ± 8.15#
Renal free iron (nmol/mg protein)	8.56 ± 2.14	25.74 ± 4.96*	23.32 ± 5.71	18.47 ± 3.63#	15.89 ± 2.7#
Plasma free iron (mmol/L)	17.04 ± 3.21	40.12 ± 5.45	31.85 ± 6.12	27.32 ± 2.96	21.87 ± 4.23
Hepatic calcium (μmol/mg protein)	82.22 ± 8.54	180.14 ± 19.05*	176.69 ± 12.41	150.48 ± 18.67#	108.21 ± 10.23#
Renal calcium (nmol/mg protein)	2.07 ± 0.3	6.98 ± 1.11*	6.83 ± 1.28	5.98 ± 0.68#	4.87 ± 1.05#
Plasma calcium (mmol/L)	0.71 ± 0.03	2.82 ± 0.45*	2.74 ± 0.25	2.08 ± 0.81#	1.32 ± 0.07#

*Note*: Animals were pretreated with various doses of SF‐AE (50, 100, and 200 mg/kg, b.w., p.o.) or vehicle (NaCl 0.9%) during 15 days. One hour after, each animal, except group 1 was received EtOH (5 mL/kg, b.w.) and after 30 min castor oil (5 mL/kg, bw., p.o.) by gavage for 2 h. The data are expressed as mean ± SD (*n* = 10).

* *p* < 0.05 compared to control group.

^#^ *p* < 0.05 compared to EtOH + CO group.

## 4 DISCUSSION

In the present research, we investigated the therapeutic efficacy of SF‐AE against hepatonephrotoxicities induced by simultaneous administration of ethanol (EtOH) and castor oil (CO). We hypothesized that EtOH/CO and SF‐AE could induce hepatonephrotoxities and would exert clearer protective actions compared to female rats.

In vitro, our phytochemical screening results using colorimetric and biochemical methods showed that SF‐AE showed an important antioxidant capacity against the DPPH^•^ radical. This potential could be explained, partly, to the diversity in phenolic composition. In this regard, our data also suggests that SF‐AE contains high contents of total polyphenols, flavonoids, and condensed tannins. However, these active biomolecules are known for their strong antioxidant potential (Choukairi et al., 2020; Khiya et al., 2021).

The analysis of SF‐AE by LC‐MS method identified 12 phenolic compounds including phenolic acids and flavonoids as catechin (+), naringin, and quercetin (Jedidi et al., 2022a). These bioactive compounds exhibit anti‐inflammatory and reactive oxygen species (ROS) scavenging properties (Marchica et al., 2018; Salaritabar et al., 2017).

In vivo, the combined effects of ethanol intoxication and castor oil poisoning were studied. The simultaneous administration firstly induced gastrointestinal ulcer and secretory diarrhea confirmed by the alteration of gastrointestinal mucosa and fluids accumulation in the small intestines (Jedidi et al., 2022a).

During this investigation, we also found that the simultaneous administration of EtOH and CO caused severe liver and kidney tissue damage. These observations are in line with results from the literature, which have shown that ricin and/or alcohol intoxications were very effective for the induction of typical injuries (Elgharbawy et al., 2021; Jedidi et al., 2022b; Kamoun et al., 2017). These are accompanied by edema and focus of necrosis and blood congestion with dilation of the centrilobular veins in the livers. For the kidneys, distensions of tubules, blood congestions, and parenchyma were detected.

The obtained results showed a significant increase in liver and kidney weights was detected in rats intoxicated with EtOH/CO when compared to the untreated group. This phenomenon has been previously showed in many researches concerning the liver and kidney size (Jedidi et al., 2022b; Selmi et al., 2015). More importantly, subacute SF‐AE pretreatment at various doses (50, 100, and 200 mg/kg, bw, p.o.) reduced histological changes, as well as the increase of weights after simultaneous administration of EtOH/CO.

Importantly, the consumption of alcohol combined with castor oil significantly increased the levels of various liver enzyme biomarkers such as AST, ALT, LDH, albumin, and direct bilirubin compared to control groups. This finding indicates cell leakage and loss of functional integrity in the liver. However, our results are in agreement with those previously conducted by Yimam et al. (2016). These transaminases are located in the cytoplasm of hepatocytes and are emptied via the circulation when the cell membrane is damaged (Adewale et al., 2014). In this context, chemical agents (EtOH + CO) have induced liver parenchymal damage and cause elevated plasma bilirubin concentration (Darbar et al., 2011; Guerra Ruiz et al., 2021), which may be explained by a malfunction of the liver and consequently a slowing down of their evacuation in the bile.

More impressively, subacute SF‐AE pretreatment protected all these deregulations. However, the results of the present study are consistent with those published by Amujiri et al. (2021) who suggested that *Psidium guajava* extract has an important potential hepatoprotective against castor oil‐induced damage. The same hepatonephroprotection role has been observed with other plant species such as *Lavandula stoechas* (Selmi et al., 2015) and *Ceratonia siliqua* (Rtibi et al., 2016).

Simultaneous acute intoxication by alcohol and castor oil induced the alteration of kidney function parameters, such as the increase of plasma urea and creatinine contents, accompanied by the reduction of plasma uric acid content, as well as the decrease in urea nitrogen. Indeed, these markers are generally used for the assessment of kidney function (Mathew, 2020).

Our results were in line with those reported in prior studies. It has also been suggested that the damage to the kidneys is more pronounced during the period of intoxication. These findings could probably be explained by their blood supply and responsibilities in the metabolic mechanism, expulsion of both chemicals and the alteration of structural integrity of nephrons (Bellassoued et al., 2018). Moreover, it has been previously suggested that the level of creatinine increased when half of the renal nephrons were damaged (Dong et al., 2014).

However, consumption of SF‐AE for 15 days significantly corrected these parameters of the renal balance. The same nephroprotective effect has been previously observed in other plant species such as *Trachyspermum ammi* (Farzaei et al., 2018), *Salvia officinlis* (Jedidi, Selmi, et al., 2022), and *Mentha piperita* (Bellassoued et al., 2018).

Interestingly, the current study showed a marked increase in total cholesterol, triglycerides, and LDL‐cholesterol levels, accompanied by a reduction in HDL cholesterol in the EtOH + CO group when compared to the control. Subacute administration of SF‐AE protected against any metabolic parameters deregulations. This observation could be explained by the modification of the hepatic cells permeability (Yousef et al., 2003). Indeed, HDL‐C can accelerate the removal of cholesterol from peripheral tissues to the liver for catabolism and excretion. It has been reported that high level of HDL can compete with LDL receptor sites on arterial smooth muscle cells and consequently inhibit LDL‐C degradation and cholesterol solubility in micelles, which may lead to retardation of cholesterol absorption (Bowry et al., 1992).

However, the protective potential of SF‐AE is in line with Duangjai et al. (2019) who showed that *Azadirachta indica* extract exerted, in vitro, a powerful lipid profile parameters regulatory activity.

The obtained results also demonstrated that SF‐AE protected against inflammation caused by EtOH combined with CO, expressed by the increase in both ALP activity and CRP level. Several studies previously proven that the consumption of ethanol and castor oil alone/combined exerted the installation of inflammatory reactions (Jedidi, Aloui, et al., 2022; Jedidi, Selmi, et al., 2022; Selmi et al., 2015). Other studies have suggested that these two agents induced the expression of other inflammatory parameters/markers. Hepato‐ and nephrotoxicity induced by alcohol, castor oil, or ricin have been shown to be accompanied by excessive production of pro‐inflammatory markers and oxidative stress (Elgharbawy et al., 2021; Liu et al., 2015). In addition, it has been suggested that nuclear factor‐κB (NF‐κB) is activated by various inflammatory stimuli such as cytokines and TNF‐α (Lennikov et al., 2018).

Interestingly, the anti‐inflammatory activity, exerted by SF‐AE, can be explained by the richness of SE‐AE in phenolic compounds which strongly inhibit the generation of pro‐inflammatory markers (cytokines) and the establishment of NF‐κB signaling. The same findings have been observed in animal models (Karunaweera et al., 2015).

To compare the individual effect of ethanol and castor oil each alone, Jedidi et al. (2022b) evaluated the effect of *Salvia officinalis* flowers decoction extract (SODE) against EtOH alone induced liver and kidney injuries. In the same respect, we recently published a book in the European University Editions (Jedidi, 2023) which focuses on the benefits of the *Salvia officinalis* leaves aqueous extract (SOLAE) on hepatorenal toxicities. The results demonstrated that the consumption of ethanol or castor oil alone induced both hepatic and renal tissue toxicities, which manifest as structural changes, disturbances in hepatic/renal function and lipid profile parameters. The two inducing agents each caused a state of oxidative stress and an inflammatory reaction. All these physiological, histological, and biochemical alterations were significantly dose‐dependently corrected way and by the bioactive molecules of the two studied extracts.

Oxidative stress refers to an imbalance between the overload of reactive oxygen species (ROS) and the antioxidant system. Living organisms protect themselves against oxidative damage through an endogenous antioxidant defense system.

In the present study, acute administration of EtOH/CO also led to a significant increase in hepatic and renal malondialdehyde (MDA), with a decrease in antioxidant enzymes activities (SOD, CAT, GPx) and non‐enzymatic antioxidant levels (GSH and –SH groups). The toxicity, caused by ethanol and ricin, induced a state of oxidative stress, which leads to cell damage, via the disruption of energy metabolism at the level of the mitochondrial membrane (Guo et al., 2014; Kumar et al., 2007).

Pretreatment with SF‐AE, at increasing doses, preserved the redox status. The antioxidative properties offered was significantly correlated to the richness of the plant aqueous extract in phenolic acids (quinic, protocatchuic, p‐coumaric, trans‐cinnamic acids) and flavonoids (catechin (+), naringin, and quercetin). These molecules are powerful antioxidants and have exerted their actions against oxidative stress following the restoration of the activities and levels of endogenous enzymatic and non‐enzymatic antioxidants (Bellassoued et al., 2018).

Finally, we tested the combined effect of these two toxic agents against certain intracellular mediators in this possible hepatorenal protection. Pretreatment with SF‐AE also corrected the increase in hepatic, renal, and plasma hydrogen peroxide (H_2_O_2_) and free iron (Fe) levels induced by simultaneous administration of EtOH and CO. However, these two molecules catalyzed, via the Fenton reaction, the production of highly reactive hydroxyl radical (OH^•^), leading to lipid peroxidation (Lyngsie et al., 2018). We also showed that intoxication with EtOH/CO elevated the level calcium. However, SF‐AE regulated the disruption of this homeostasis. Furthermore, it is known that ROS overproduction can increase the levels of this element in different cell types (Hempel & Trebak, 2017). However, the bioactive molecules of SF‐AE appear to act through a complex mechanism that prevents calcium accumulation in liver and kidney cells. This finding can also be explained by the fact that SF‐AE can cause inhibition of Ca^2+^ influx and nuclease activity (Hernandez‐Muñoz et al., 2000).

The same protective effect against the increase of intracellular mediator's levels has already reported in prior studies for other plant extracts such as *Lavandula stoechas* (Selmi et al., 2015) and *Ceratonia siliqua* (Rtibi et al., 2016).

## 5 CONCLUSION

Our findings clearly indicated that SF‐AE can ameliorate combined EtOH‐ and CO‐mediated hepatonephrotoxicity. Simultaneous acute EtOH/CO intoxication induced oxidative stress. Furtherly, this status causes liver and kidney tissues damage, transaminases deregulations, lipid profile alteration, subsequently, the installation of inflammatory reaction in EtOH + CO group. Our data evidenced that SF‐AE effectively inhibited oxidative stress, counteracted intracellular mediators increase and the levels of inflammatory markers. The obtained results confirmed the use of sage extract in Tunisian traditional medicine for the treatment and/or management of digestive system disorders, such as liver and kidney pathologies and suggest its potential as an additive in the food and pharmaceutical industries.

## AUTHOR CONTRIBUTIONS

**Saber Jedidi** and **Houcine Selmi** performed the experiments, analyzed the data, and wrote the article. **Foued Aloui** participated in the processing and analysis of the data. **Kais Rtibi** participated in conducting various analyses such as phytochemical, animal treatments, and sacrifices. **Hichem Sebai** participated in the data processing and the design of the experiments. All authors read and approved the final manuscript.

## FUNDING INFORMATION

The author(s) received no financial support for the research, authorship, and/or publication of this article.

## CONFLICT OF INTEREST STATEMENT

The authors declare that there are no competing interests.

## ETHICS STATEMENT

All maintenance and sacrifice procedures were used following the local ethics committee of Tunis University of the use and care of animals and in accordance with the NIH recommendation. The protocol was approved by the “Comité d'Ethique Bio‐medicale (CEBM)” (JORT472001) of the “Institut Pasteur de Tunis.”
